# Hypertrophic Stimulation Increases β-actin Dynamics in Adult Feline Cardiomyocytes

**DOI:** 10.1371/journal.pone.0011470

**Published:** 2010-07-12

**Authors:** Sundaravadivel Balasubramanian, Santhosh K. Mani, Harinath Kasiganesan, Catalin C. Baicu, Dhandapani Kuppuswamy

**Affiliations:** 1 Cardiology Division, Department of Medicine, Gazes Cardiac Research Institute, Medical University of South Carolina, Charleston, South Carolina, United States of America; 2 Ralph H. Johnson Department, Veterans Affairs Medical Center, Charleston, South Carolina, United States of America; Harvard Medical School, United States of America

## Abstract

The myocardium responds to hemodynamic stress through cellular growth and organ hypertrophy. The impact of cytoskeletal elements on this process, however, is not fully understood. While α-actin in cardiomyocytes governs muscle contraction in combination with the myosin motor, the exact role of β-actin has not been established. We hypothesized that in adult cardiomyocytes, as in non-myocytes, β-actin can facilitate cytoskeletal rearrangement within cytoskeletal structures such as Z-discs. Using a feline right ventricular pressure overload (RVPO) model, we measured the level and distribution of β-actin in normal and pressure overloaded myocardium. Resulting data demonstrated enriched levels of β-actin and enhanced translocation to the Triton-insoluble cytoskeletal and membrane skeletal complexes. In addition, RVPO *in vivo* and *in vitro* hypertrophic stimulation with endothelin (ET) or insulin in isolated adult cardiomyocytes enhanced the content of polymerized fraction (F-actin) of β-actin. To determine the localization and dynamics of β-actin, we adenovirally expressed GFP-tagged β-actin in isolated adult cardiomyocytes. The ectopically expressed β-actin-GFP localized to the Z-discs, costameres, and cell termini. Fluorescence recovery after photobleaching (FRAP) measurements of β-actin dynamics revealed that β-actin at the Z-discs is constantly being exchanged with β-actin from cytoplasmic pools and that this exchange is faster upon hypertrophic stimulation with ET or insulin. In addition, in electrically stimulated isolated adult cardiomyocytes, while β-actin overexpression improved cardiomyocyte contractility, immunoneutralization of β-actin resulted in a reduced contractility suggesting that β-actin could be important for the contractile function of adult cardiomyocytes. These studies demonstrate the presence and dynamics of β-actin in the adult cardiomyocyte and reinforce its usefulness in measuring cardiac cytoskeletal rearrangement during hypertrophic stimulation.

## Introduction

Actin is a ubiquitously expressed and highly conserved cytoskeletal protein essential for numerous cellular processes including cell attachment, spreading, cytokinesis, vesicular trafficking, transcription, translation, and ion transport [1]. Of the six mammalian actin isoforms (α-cardiac, α-skeletal muscle, α−smooth muscle, β-cytoplasmic, γ-cytoplasmic and γ-enteric), five are expressed in the embryonic myocardium while α-actin predominates into adulthood. However, the expression pattern mimicking the fetal stages can be observed both in adult cardiomyocytes undergoing hypertrophy or in culture in the presence of various hypertrophic stimuli [1]. β-Actin is expressed in all four chambers of the heart as well as in cultured adult cardiomyocytes [2], [3]. Overexpression of β-actin promotes cell spreading in many cell types, and in dilated cardiomyopathy elevated levels of β-actin have been reported [4]. Transfection of HeLa cells with siRNA for β- and γ-actin causes cell blebbing suggesting cytoskeletal collapse and apoptotic cell death [5]. While a β-actin knockout mouse is not available, the skeletal muscle-specific deletion of γ-actin in mice leads to compromised contractility due to disorganization of myofibrils at costameres where β-actin is suggested to play a redundant role in myofibrillogenesis [6]. β-actin in conjunction with nonmuscle myosin II regulates contractile processes in non-myocytes. With the application of the fluorescent recovery after photobleaching (FRAP) technique, it has been shown in skeletal muscle cells that various cytoskeletal proteins are associated dynamically with Z-discs [7]. β-actin-containing molecular complexes are critical in cytoskeletal rearrangement upon cell shape modulating signals. For example, in osteoclasts, β-actin is known to organize the podosomes during osteoclasts motility [8]. Although cardiomyocytes express β-actin in considerable amounts, whether it participates in cytoskeletal rearrangement upon hypertrophic stimulation remains poorly understood.

Hemodynamic overload during physiological and pathological events initiates a process of cardiac growth. The current understanding is that physiological hypertrophy is initiated as part of an adaptive mechanism whereas pathological hypertrophy due to cardiac stress culminates in maladaptive myocardial remodeling [9]. Several signaling processes including fetal gene re-expression, activation of protein translation, increase in mass, and enlargement in cell size/volume have been identified as markers of hypertrophy. Of the multiple processes leading to growth, the one that contributes to cytoskeletal rearrangement is considered as a hallmark of hypertrophy.

In non-myocytes, actin carries out a vast number of functions including migration, protein/mRNA transport, transcriptional regulation and cytoskeletal architecture. Actin exists both as a globular (G) and a filamentous (F) forms due to a dynamic polymerization and depolymerization processes. This polymerization/depolymerization cycle is essential for many of the aforementioned actin functions and is governed by a plethora of actin-binding proteins (ABPs). In response to extracellular stimuli, ABPs regulate actin dynamics in various steps such as actin filament nucleation, elongation, branching, cross linking, capping, severing, and monomer sequestration. One of the major transducers of extracellular signals to the ABPs is the small GTPases of the Rho family, namely, Rho, Rac and cdc42. Upon activation, these GTPases regulate the function of ABPs such as Arp2/3, WASP, and cofilin as well as influence the dynamics of actin polymerization [10]. Although many of these ABPs are present in cardiac myocytes, their precise role in the maintenance of sarcomeric structure is largely unknown. The cytoskeletal actin isoforms (other than cardiac α− actin found in contractile myofilaments) are found in costameres and Z-disc areas and perform functions similar to non-myocytes, namely vesicle trafficking, and ion channel tethering [11]. While γ-actin has been proposed to be at the costameres of skeletal myocytes [12], [13], the identity of an actin isoform with similar function in cardiac myocytes is lacking. β-actin along with the γ- actin isoform is expressed in pre-differentiated striated muscle cells. However, in adult myocytes its expression level is low where it is localized to costameres, neuromuscular junctions, T-tubules, and sarcoplasmic reticulum. γ-cytoplasmic actin is shown to be present at the costameric filament network that connects the peripheral sarcomeres to the dystroglycan complex at the sarcolemma. Several studies report the existence of spatiotemporal distribution of γ-actin isoforms in adult skeletal muscle cells. However, this isoform or the one with a similar function and localization has not yet been reported for cardiac myocytes [11].

Since non-myocytes utilize β-actin for cytoskeletal rearrangement and since β-actin is expressed in adult cardiomyocytes, we hypothesized that β-actin localizes at the Z-discs of adult cardiomyocytes and its dynamics is altered by hypertrophic signals. Our results suggest that β-actin is present in Z-discs, costameres, and intercalated disc regions and that β-actin's dynamics is amenable to alterations upon hypertrophic stimulation.

## Materials and Methods

### *In vivo* hypertrophy model

Feline model of right ventricular pressure overload (RVPO) was used to induce hypertrophy for 48 h as described before [14]. Pressure overload of the right ventricle (RV) was induced in felines (n = 6) by partially occluding the pulmonary artery with a 3.2-mm internal diameter band. RV pressure was more than doubled, while systemic arterial pressure remained unaltered. Controls consisted of sham-operated felines (n = 6) submitted to thoracotomy and pericardiotomy without hemodynamic intervention, or the normally loaded left ventricle (LV) from each animal having RVPO. All operative procedures were carried out in cats weighting 2.6–3.9 kg under full surgical anesthesia with meperidine (2.2 mg/kg intramuscularly), acepromazine maleate (0.25 mg/kg intramuscularly), and ketamine HCl (50 mg/kg intramuscularly) as per approved protocols of the institutional committee. After completion of pressure overloading, the animal was heparinized and placed on oxygen. A left thoracotomy was performed, and the heart was rapidly removed, placed in cold buffer solution, and weighted. The aorta was then cannulated; the coronary arteries were perfused with phosphate-buffered saline and the free walls from the LV and RV were removed for immediate processing.

### Isolation of primary adult cardiomyocytes

Adult feline cardiomyocytes were isolated to >95% purity as published previously [15]. In the present study, we used glass-bottom dishes (MatTek) coated with laminin for culturing cardiocytes in Piper's medium, which was prepared in M199 cell culture medium (Invitrogen) containing the following additional ingredients: 2% bovine serum albumin, 5 mM creatine (Sigma), 2 mM L-carnitine (Sigma), 5 mM taurine (Sigma), 0.25 mM L-ascorbate (Sigma), 10 µM AraC, 200 units/ml penicillin and 200 µg/ml streptomycin (Invitrogen). After plating the freshly isolated adult cardiomyocytes overnight, they were infected with 10 MOI each of Tet-Off and β-actin-GFP adenoviruses and cultured in Piper's medium for additional 24–36 h. Then the cells were subjected to FRAP in the presence or absence of agonist stimulation.

### Determination of F-actin/G-actin ratio of β-actin isoform

Separation of polymeric F-actin and monomeric G-actin pools of β-actin isoform from tissue or cells was performed as published previously [16] using a commercial kit (Cytoskeleton, Inc). Briefly, cells or tissue was extracted using the F-actin stabilization buffer provided in the kit. After centrifugation at 100,000x*g* for 1 h at 37°C, the F-actin pellet was separated from the G-actin supernatant. The supernatant was collected and the remaining pellet was treated with cytochalasin D to depolymerize F-actin. Both the G- and F-actin pools were reconstituted to the same volume and then analyzed by Western blotting using anti-β-actin antibody.

### Adenoviruses

We received β-actin-GFP and Tet-Off adenoviruses from Dr. Scott Soderling (Duke University, North Carolina) and YFP-β-actin adenovirus from Dr. Pietro DeCamilli (Yale University, Connecticut) respectively. Dominant negative Rac1 T17N (Rac N17) adenovirus was constructed using pAdEasy system as we described previously [17] using Rac1 (T17N)-PUSEamp construct (Millipore).

### Antibody transduction and immunocytochemistry

For transducing antibodies into adult cardiomyocytes, we used GenomONE-CABEX reagent that uses the envelope of hemagglutinating virus of Japan (HVJ; an inactive form of Sendai virus) according to the manufacturer's instructions (Cosmobio, USA, #AB001EX). For immunocytochemical staining of cells grown on glass cover slips, either normal cardiomyocytes or cardiomyocytes transduced with β-actin antibody via HVJ were fixed in 1% paraformaldehyde for 10 min. Followed by washing in phosphate buffered saline (PBS) thrice each for 5 min, the cells were permeabilized with 0.05% Triton X-100 in PBS for 5 min and washed with PBS (3×5 min). After blocking with 10% normal donkey serum in PBS for 1 h at room temperature, the coverslips were treated with the specified primary antibodies (overnight at 4°C) followed by fluorescent-labeled secondary antibody (2 h at room temperature). After washing (5×5 min) and mounting, the cover slips were imaged using LSM 510 (Zeiss) microscope. Freshly frozen cryosectioned tissue or fixed cells were used for confocal immunostaining as described previously [14], [18].

### Cell and tissue lysate preparation and Western blotting

Fresh ventricular samples were extracted with Triton buffer as described previously [14] to obtain Triton X-100-soluble (Sol), cytoskeleton (Triton X-100-insoluble, 10,000 x *g* pellet; CSK), and membrane skeleton (Triton X-100-insoluble 100,000 x *g* pellet; MSK).

### Cell shortening

Measurement of cell shortening in adult feline cardiomyocytes upon electrical stimulation was carried out using the IonOptix system (IonOptix, Milton, MA) as described previously [19]. Isolated cardiomyocytes were placed at room temperature (25°C) in a custom made study chamber and viewed with a Nikon inverted microscope. The cell image was collected by an X40 objective lens, and then transmitted to a MyoCam CCD video camera. Cardiomyocytes contracted in response to a 10% over-threshold 1 Hz/5 ms electrical signal provided by a Grass88 electrical stimulator and a set of two platinum electrodes placed in the study chamber. Cell length and contraction amplitude were recorded in real time with a video edge detector and specialized data acquisition software (SoftEdge and IonWizard; IonOptix). The CCD camera was adapted to acquire images at a 240 Hz frame rate and 4.2 ms time resolution. The signal-to-noise ratios were significantly improved by averaging 10 sequential runs. Calibration of the system was accomplished with a micrometer and data acquisition software. Data are presented as Mean±SEM. Statistical comparisons among groups were performed by Student's t-test. p<0.05 was considered significant.

### Fluorescence recovery after photobleaching (FRAP)

Live adult cardiomyocytes grown on glass-bottom dishes coated with laminin were used for FRAP studies as published for skeletal myocytes [7]. The cells were maintained at 37°C and 5%CO_2_ during live microscopic analysis using LSM 510 (Zeiss) confocal microscope with 63X oil immersion objective and the LSM 510 Meta software. The excitation wavelength is 488 nm (long pass 505 nm emission filter) for the β-actin-GFP construct. All images were obtained at low laser intensity to minimize laser-induced toxicity. The region of interest in cardiomyocyte is selected (normally 4–6 sarcomeres) and bleached at 100% laser intensity at 488 nm (50 iterations). After photobleaching, the recovery is recorded for up to 30 min to 1 h at 1 min intervals. The fluorescent intensity in the region of interest over time is saved as a Table using the software. From this, the percent recovery of fluorescence intensity is calculated for each time point. Kaleidagraph is used for curve fitting by two exponential expression using the formula published by Wang *et al* [7] for skeletal muscle Z-discs: R  =  M1(1-exp(-k1t)) + (M2(1-exp(-k2t) where R is the relative post-bleach recovery of fluorescence intensity at time t and k1 and k2 are the two rate constants of recovery. The t½ of recovery (time taken for half maximal recovery) is calculated by ln2/k. For each t½ there was a minimum of 10 measurements and the value was expressed as Mean±SD.

## Results

**Figure 1:**
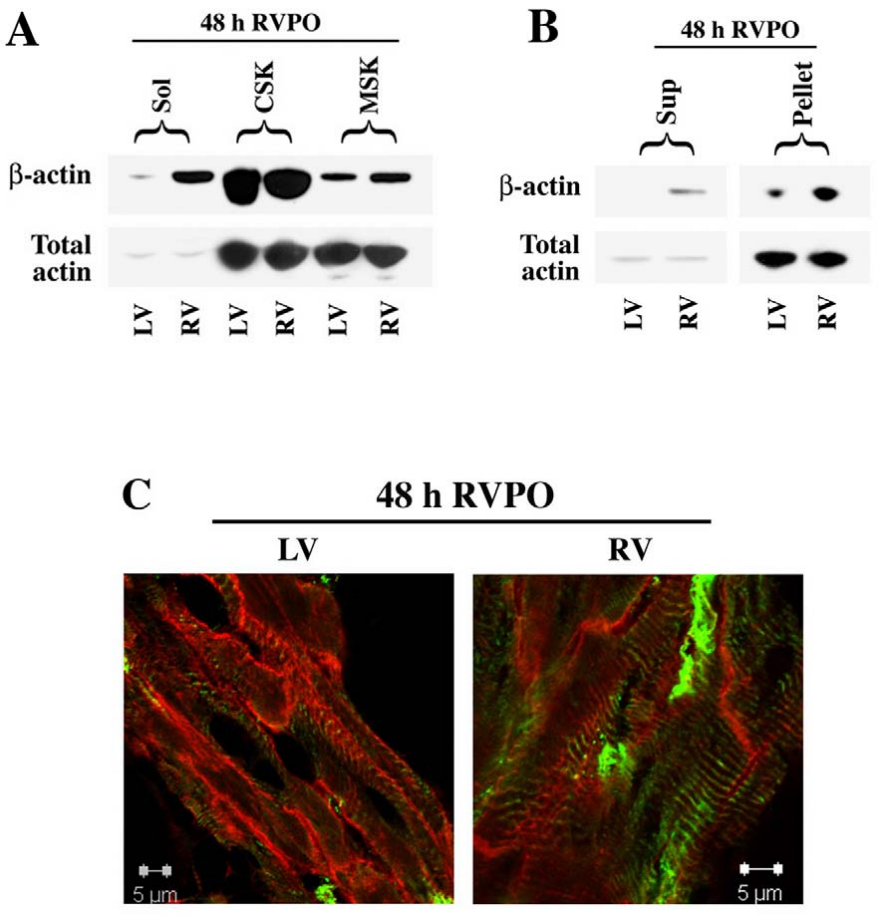
β-actin is enriched and localized to cardiac cytoskeleton during pressure overload induced hypertrophy. **A**) Normally loaded LV and pressure overloaded RV tissues from adult felines that underwent right ventricular pressure overload hypertrophy (RVPO) for 48 h were extracted into Triton-soluble, Triton-insoluble low-spin cytoskeletal (CSK) and Triton-insoluble high-spin membrane skeletal (MSK) fractions. The tissue fractions were separated by SDS-PAGE and Western blotted with indicated antibodies. **B**) LV and RV tissues from feline RVPO myocardium were fractionated into F- and G-actin as described under Materials and Methods. The pellet (corresponding to F-actin) and the Supernatant (Sup; corresponding to G-actin) were analyzed by Western blot using indicated antibodies. **C**) LV and RV tissue samples were cryosectioned into 12 µm slices and immunostained with β-actin (green) and α−actinin (red) antibodies and analyzed using a confocal microscope. Scale bar  = 5 µm.

A feline RVPO model was used to study the role of β-actin in cardiac hypertrophy, upon which the pulmonary artery was ligated with a 3.2 mm internal diameter band which doubles the RV pressure while the LV pressure remains unchanged [20]. As reported earlier, when compared to the normally loaded same animal LV or Sham control LV and RV, the pressure overloaded RV exhibits classic signs of hypertrophy including fetal gene expression, protein synthesis, myocyte cross sectional area increase, and integrin-mediated tyrosine kinase signaling [14]. While it is conceivable that cardiomyocyte structural alterations such as growth and remodeling require coupling with actin dynamics, any evidence that a specific isoform of actin is involved in such phenotypic modification in cardiomyocytes has yet to be established. As a first step to link the involvement of β-actin in cardiac hypertrophy, we fractionated both LV and RV into Triton-soluble (sol), Triton-insoluble (low-spin cytoskeletal), and Triton-insoluble (high-spin; membrane skeletal) fractions and analyzed by Western blotting. As shown in Figure 1A, β-actin which is enriched in the Triton-soluble RV, but not in the corresponding LV tissue. In addition, the increased β-actin level is associated with enhanced recruitment of β-actin to the membrane skeletal fraction. This suggests that β-actin that is enriched during 48 h of hypertrophic induction as a result of activation of multiple signaling cascades [9] is found in the soluble (mostly cytosolic) as well as in the Triton-insoluble membrane skeletal fractions. Total actin including α−sarcomeric actin and any other isoforms present in the heart are shown as a loading control which does not exhibit any noticeable change. This could be due to the fact that the cardiac α−actin constitutes the majority of the total actin in the heart, and any change in the other minimally expressing actin isoforms such as β-actin could be masked by the cardiac α−actin isoform when a non isoform selective total actin antibody is used in Western blotting. Next, to test if hypertrophy induces the β-actin polymerization, a surrogate marker for β-actin dynamics, we used a commercial kit that allows the separation of monomeric G-actin (supernatant) and polymeric F-actin (pellet) in LV and RV tissues from a 48 h RVPO feline. The supernatant corresponding to G-actin and the pellet corresponding to F-actin were then immunoblotted for β-actin specific antibody. Figure 1B suggests that the β-actin level is enriched in the RV supernatant (G-actin), further confirming the RV soluble data presented in Figure 1A. In addition, the enriched β-actin is present in the filamentous F-actin form (pellet), indicating that β-actin polymerization is upregulated in 48 h pressure overloaded ventricle. To localize β-actin in the pressure overloaded myocardium, we performed immunohistochemical staining. Even though a considerable amount of β-actin is found to be expressed in the myocardium as revealed by Western blots (Figure 1A, Figure 1B), immunohistochemical staining of β-actin was weak (Figure 1C) despite the use of various antibodies and fixation protocols. However, the IHC data show that in RV, the enriched β-actin is predominantly localized in cardiomyocytes. These data suggest that the β-actin level is increased specifically in the pressure overloaded RV and is present as the polymerized F-actin form. This localization in adult cardiomyocytes indicates a possible role in adult cardiomyocyte hypertrophy.

**Figure 2:**
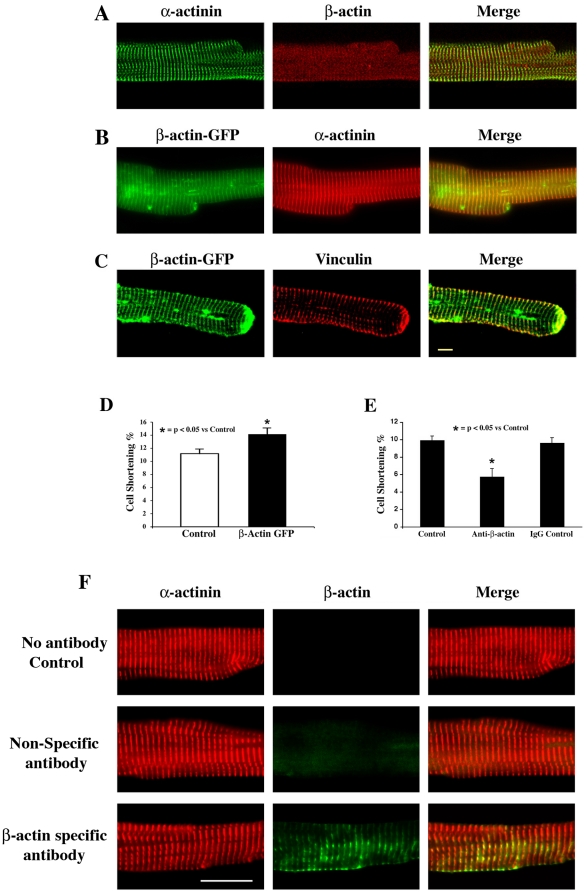
β-actin is localized to adult cardiomyocyte cytoskeleton and contributes to contractility of cardiomyocytes. **A**) Adult cardiomyocytes cultured on laminin coated cover slips were fixed and stained for β-actin (red) along with α−actinin (green), and the image was recorded using a confocal microscope. **B**) Adult cardiomyocytes cultured on laminin coated cover slips were infected with β-actin-GFP adenovirus (MOI = 10). After 24–36 h of infection, the cells were fixed and stained using α−actinin antibody. The GFP fluorescence corresponding to β-actin and red fluorescence corresponding to α−actinin staining were recorded by confocal microscopy. **C**) Cells were processed as in B above except for they were costained for vinculin localization (red). **D**) Adult cardiomyocytes were infected with an equal MOI of β-gal (Control) or β-actin-GFP adenovirus and the proteins were allowed to express for 24–36 h. After this period, the cells were subjected to electrical stimulation, and cell shortening measurement was performed as described under Materials and Methods. The percent shortening is recorded for 15 cells in each condition and the experiment was repeated at least thrice. **E**) Adult cardiomyocytes were transduced with either β-actin antibody or control IgG using HVJ as described under Materials and Methods. After overnight incubation with the transduction mixture, cells were then subjected to cell shortening measurement as described under Materials and Methods. The percent shortening is recorded for 15 cells in each condition and the experiment was repeated at least thrice. **F**) Adult feline cardiomyocytes grown on laminin-coated cover slips were transduced with either β-actin antibody or control IgG using HVJ envelope as described under Materials and Methods. After overnight incubation with the transduction mixture, the cells were fixed and incubated with rabbit α−actinin primary antibody. Subsequently the cells were immunostained with Alexa 568– labeled anti-rabbit IgG to stain for α−actinin and, Alexa 488– labeled anti-mouse IgG to detect β-actin antibodies that were transduced into the cells by HVJ envelope. Scale bar  = 5 µm.

Next, to study the role of β-actin at the level of adult cardiomyocytes, we used isolated adult cardiomyocytes from normal felines and cultured them in serum-free medium. When these cells were fixed and analyzed for β-actin, there was only minimal reactivity (Figure 2A) as compared with α−actinin costaining. To study the effect of increased β-actin expression, we adenovirally expressed β-actin tagged to GFP at the C-terminus (β-actin-GFP) at an MOI of 10. After 24–36 h of infection, cells were fixed and stained for α−actinin and vinculin separately. As seen in Figure 2B, the adenovirally expressed β-actin-GFP is localized at the Z-discs of adult cardiomyocytes, similar to α−actinin staining. In addition, β-actin-GFP is localized at the costameres and at the cell terminus as seen by vinculin colocalization (Figure 2C). The expression of β-actin-GFP at 10 MOI level did not affect the morphology of adult cardiomyocytes. Since β-actin levels are enriched during hypertrophic induction and are associated with both Z-discs and costameres, we hypothesized that β-actin may improve the contractility of cardiomyocytes. For this investigation, we subjected adult cardiomyocytes plated on laminin, with and without β-actin-GFP expression, to electrical stimulation. While control cells (β-gal infected) exhibited 11.2±0.7% contraction, cells expressing β-actin-GFP exhibited 14.1±1.0% contractility (Figure 2D). To further establish that β-actin is directly involved in cell contractility we neutralized endogenous β-actin in cultured cardiomyocytes by transducing β-actin specific antibodies through HVJ-mediated delivery [21]. Cardiomyocytes transduced similarly with non-specific IgG served as controls. After overnight incubation with the neutralizing antibody, cells were subjected to electrical stimulation for contractility measurement. Figure 2E reveals that blocking with β-actin but not with non-specific IgG results in a significant reduction in contractility. To confirm that the transduced β-actin antibody bound only the β-actin and not the cardiac α−actin, we fixed the antibody transduced cells and stained with fluorescent labeled secondary antibody specific for the transduced primary antibody. All the fixed cells were stained for α−actinin. Figure 2F shows that cardiomyocytes transduced with either no antibody (top row) or a non-specific IgG (middle row) did not stain for β-actin. However, cardiomyocytes transduced with β-actin antibody were detected by the subsequently added fluorescent labeled secondary antibody (bottom panel). This result not only suggests that the contractility of β-actin antibody-transduced cardiomyocytes was impaired (Figure 2E) because of specific binding of the antibodies to β-actin, but also reiterates that the localization of β-actin is in the Z-discs and costameres as assessed by β-actin-GFP expression. Together these data suggest that β-actin upregulation during hypertrophy could play an important role in enhancing the contractility of cardiomyocytes.

**Figure 3:**
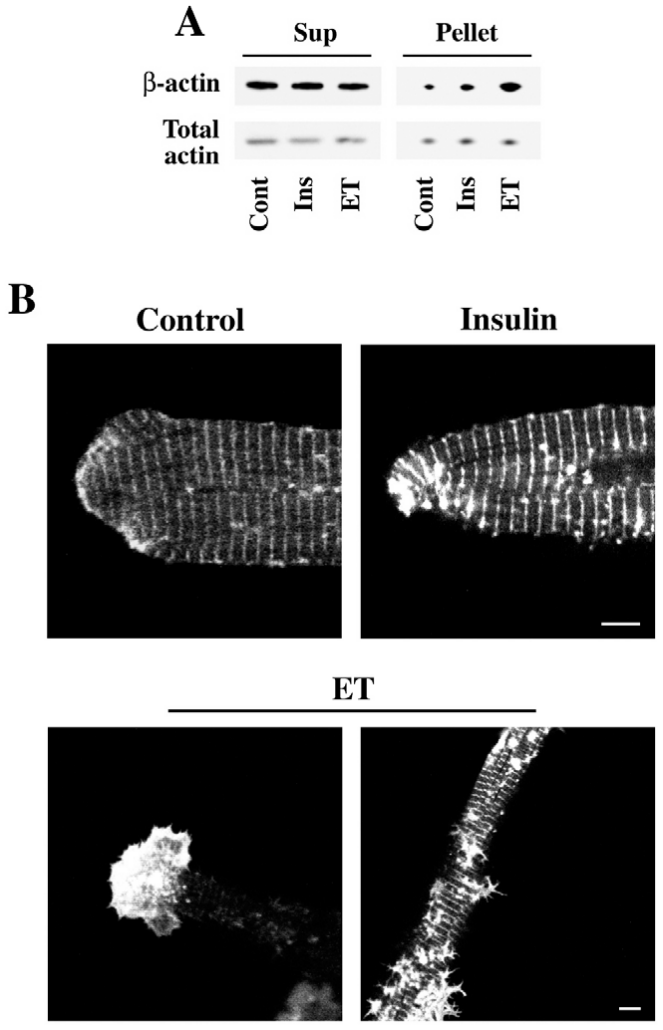
β-actin cytoskeletal reorganization during hypertrophic stimulation in cultured cardiomyocytes. **A**) Adult cardiomyocytes cultured on laminin-coated dishes were treated with 200 nM endothelin (ET) or 100 nM insulin (Ins) for 30 min. The cells were lysed and subjected to F-actin (Pellet) and G-actin (Sup) fractionation as described under Materials and Methods. Both these fractions were analyzed by Western blotting using indicated antibodies. **B**) Adult cardiomyocytes infected with β-actin-GFP were treated with insulin (100 nM) or endothelin (200 nM) for 48 h and imaged at various Z-planes using a confocal microscope. Scale bar  = 5 µm.

Next, in order to further understand the role of β-actin in adult cardiomyocytes during hypertrophy, we treated adult cardiomyocytes in culture with endothelin for 30 min and then fractionated the lysates into F- and G- actin fraction similar to the experiment shown in Figure 1B. Results in Figure 3A indicate that hypertrophic agonists, both ET and insulin, mobilized β-actin into the filamentous F-actin form suggesting that hypertrophic agonists induce β-actin polymerization. To test if such a polymerization induces any structural modifications, we treated adult cardiomyocytes with ET or insulin for 48 h and imaged them along the Z-axis using confocal microscopy. Results indicate that the β-actin-rich membrane protrusions were stronger at the cell terminus (Figure 3B, bottom left) as well as at the cell-substratum interface at regions corresponding to costameres (Figure 3B, bottom right). These actin-rich protrusive structures were more prominent in ET-treated cells than in insulin-treated cells. However, it should be noted that these protrusive structures are a phenomenon only in cultured adult cardiomyocytes and may not be an *in vivo* behavior during cellular hypertrophy [22], [23]. Together these results indicate that β-actin polymerization is induced upon hypertrophic stimulation.

**Figure 4:**
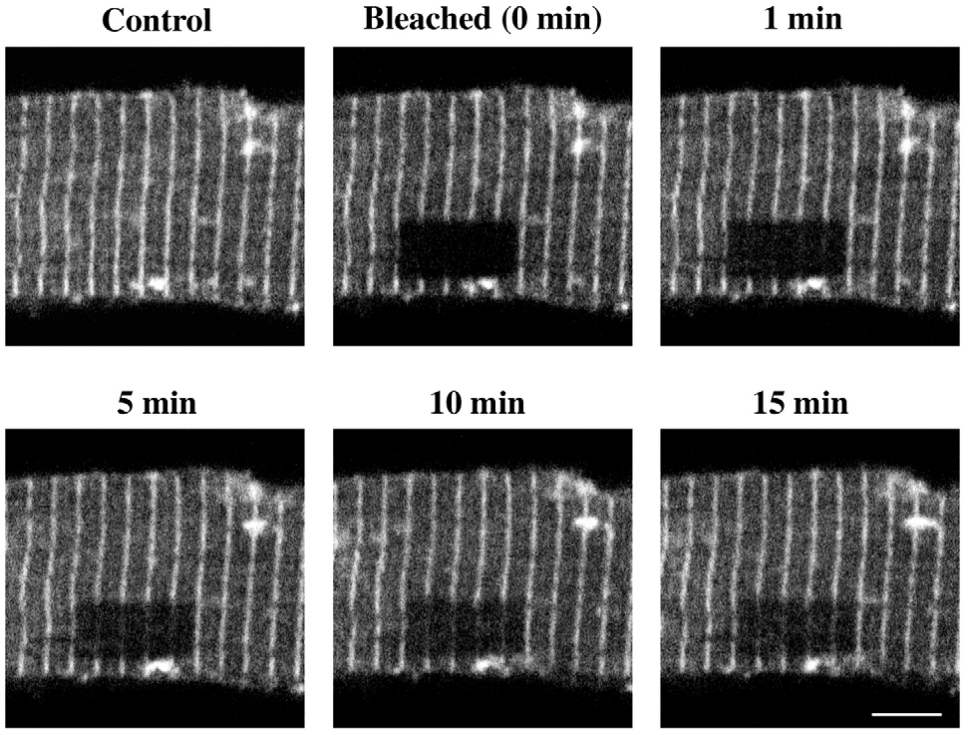
Adult cardiomyocyte β-actin dynamics measurement by using fluorescence recovery after photobleaching. Adult cardiomyocytes were infected for 24–36 h with β-actin-GFP adenovirus and subjected to FRAP analysis as described under Materials and Methods. This figure shows the prebleach (control), bleached area (black rectangle at time 0) and the subsequent recovery of fluorescence within the bleached rectangular area (1, 5, 10 and 15 min). The recovery of fluorescence within this rectangular area is measured to calculate the t½ value as presented in Table 1. Scale bar  = 5 µm.

Since we observe β-actin to exhibit specific localization in Z-discs of adult cardiomyocytes and since β-actin polymerization/depolymerization dynamics can be followed real-time using video microscopy as shown in other cell types [7], [8], we applied this approach to study the dynamics of β-actin in Z-discs. In these experiments, we expressed β-actin-GFP in cultured adult cardiomyocyte for 24–36 h and subjected them to FRAP analysis as described [7]. A rectangular area covering a portion of 4–6 sarcomeres was chosen and bleached. The recovery of β-actin-GFP fluorescence in the bleached area was monitored by imaging the cell at various time intervals (Figure 4). From the recovery of fluorescence data over time, the t½ (the time taken for half-maximal recovery) was deduced as described in Materials and Methods. Table 1 shows the t½ calculated for cells treated with insulin and endothelin for 30 min. The reduction in t½ under these conditions suggests that both endothelin and insulin enhance the β-actin dynamics upon hypertrophic stimulation.

**Table 1:** β-actin dynamics as measured by fluorescence recovery after photobleaching in adult cardiomyocytes expressing β-actin-GFP.

	t1/2 (seconds)	
	Fast	Slow
**Control**	19.0±5.4	451.6±75.3
**Insulin**	2.9±0.8*	38.8±12.0*
**ET-1**	8.8±2.4*	85.6±14.0*

Cells (n = 10) were treated with endothelin (200 nM) or insulin (100 nM) for 30 min and subjected to FRAP study. From the recovery of fluorescence the t½ was calculated as described under Materials and Methods.

**Figure 5:**
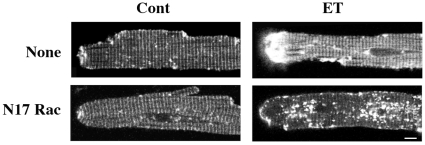
Perturbation of β-actin dynamics by dominant-negative Rac1 (N17 Rac) expression. **A**) Adult feline cardiomyocytes were infected with both β-actin-GFP and Rac N17 adenoviruses for 36 h and then stimulated with 100 nM ET for 48 h. Cells were then imaged using confocal microscopy. Scale  = 5 µm.

Finally, since Rac1 is known to mediate cytoskeletal changes and hypertrophy [24], we tested if FRAP studies with β-actin-GFP could be used as a tool to measure β-actin dynamics by doubly infecting adult cardiomyocytes with β-actin-GFP and Rac N17 adenoviruses. These studies (Figure 5) show that while ET stimulation, when compared to untreated control, caused β-actin rich membrane protrusions, this effect was suppressed in N17 Rac1 expressing cardiomyocytes. In addition, measurement of t½ of β-actin recovery by FRAP showed a reduced reassembly rate in Z-discs of N17 Rac expressing cells (Table 2). These results suggest that the measurement of β-actin dynamics could be used as a tool to test the role of various signaling pathways involved in cytoskeletal rearrangement.

**Table 2:** Table showing β-actin dynamics changes upon dominant negative Rac1 expression.

	t_1/2_ (seconds)	
	Fast	Slow
**Control**	19.5±2.3	445.7±65.2
**ET-1**	10.1±2.8*	176.9±15.7*
**N17**	61.8±10.5*	1484±197.7*
**ET-1 + N17**	15.3±2.9^#^	621.8±106.2^#^

FRAP experiments were performed in adult cardiomyocytes co-expressing the β-actin-GFP and N17 Rac following their treatment with +/− 100 nM ET. Analyses were made in at least 10 independent cells for statistical evaluation. Values are expressed as Mean ± S.D. *p<0.05 when compared to control. ^#^p<0.05 when compared to ET-1 treated cells.

In summary, the present study using GFP-tagged β-actin expression as well as β-actin antibody labeling in adult cardiomyocytes provides evidence that β actin is present in the Z-discs, costameres, and intercalated discs of adult cardiomyocytes. Our studies also suggest the functional significance of β-actin in adult cardiomyocytes by showing compromised contractility of cardiomyocytes upon neutralization of β-actin with specific antibodies. Furthermore, we demonstrate that actin monomers in Z-discs are dynamically in exchange with the cytoplasmic actin pool, the rate of which is enhanced upon hypertrophic stimulation in cultured cardiomyocytes. This notion is supported by the increased F-actin filaments of the β-actin isoform *in vivo* in a cardiac hypertrophy model. This study also suggests the utility of FRAP as a tool for following cytoskeletal rearrangement during cardiac hypertrophy.

## Discussion

The significance of cytoskeletal elements, in particular β-actin, involved in cardiomyocyte morphological rearrangement upon hypertrophic stimulation is not completely understood. Our results indicate that β-actin could play a role during cardiac hypertrophy-induced cytoskeletal rearrangement of adult cardiomyocytes. Both increased levels of β-actin and the associated contractility of cardiomyocytes could be expected to benefit hypertrophying myocardium. During ventricular hypertrophic stimulation in rats, Stilli *et al* [25] found that the expression of α−skeletal muscle actin was increased to maintain cardiac contractility and thus the mechanical performance. Although the changes associated with β-actin were not reported, this study using an infrarenal aortic banding model showed α−smooth muscle actin expression at the level of arterial vessels in prolonged hypertensive rats. A recent study [26] using a rat cardiac hypertrophy model revealed that there is a switch in actin isoforms during transition from compensated hypertrophy to decompensated hypertrophy (heart failure). This group has reported that α−skeletal actin was overexpressed during the initial stages of compensated hypertrophy whereas α−smooth muscle actin was overexpressed during the decompensated phase. While details about the changes in β-actin level were not addressed in this study, there are other reports suggesting an increased expression of β-actin during hypertrophic cardiomyopathy [4]. Gene expression profiling of hypertrophic cardiomyopathy revealed the overexpression of various cytoskeletal proteins including β-actin, α−actin, tropomyosin, fibronectin, calpain-1, and β-myosin heavy chain [4]. Therefore, our observation that β-actin is overexpressed during initial stages of pressure overload hypertrophy assumes relevance.

In neonatal rat ventricular myocytes, the expression of β-actin was found to be localized to the cell periphery exhibiting marked filopodial extensions [27]. In adult rat cardiomyocytes, forced expression of β-actin has been shown to induce peripheral actin localization with altered cellular architecture [23]. Our results show that the adenovirally expressed β-actin-GFP is associated with Z-discs and costameres. The presence of actin binding proteins such as various tropomyosin isoforms, non-muscle myosin heavy chains (NMM), and Rho family GTPases at the Z-disc area in skeletal muscle further suggests the possible existence of an actin isoform other than the cardiac α-actin filaments in muscle cells [11]. However, the identity and localization of non-sarcomeric actin isoform(s) has been lacking in adult cardiomyocytes [11]. Our study provides the evidence that β-actin is one such isoform which might play a role in the organization of Z-discs by undergoing alterations in β-actin filament length. This scenario could be analogous to the recently proposed α-actin filament length regulation achieved by capping proteins such as CapZ and tropomodulin [28]. Similar proteins could exist in the Z-discs for the regulation of β-actin isoform as well.

Torsional forces applied onto the integrin-ECM linkage sites during events such as PO could alter the composition and function of costameric complexes in order to both adapt to the changes in contractility and maintain structural integrity of the cell. In neonatal cardiomyocytes, applying a mechanical strain results in an increase in the number of sarcomeres added throughout the cell length and there is more sarcomeres added at the intercalated disc region [29]. Several labs, including ours, have established a role for integrin signaling in cardiac hypertrophic growth, survival, and structural integrity [14], [17], [30], [31], [32], [33], [34], [35], [36], [37]. Previous studies define the presence of costameres in cardiomyocytes [27], [38] and skeletal myocytes [6], [39]. Recent studies relate cardiac costameres to signal via FAK, PYK2, ILK, and RACK1 for concomitant cytoskeletal rearrangement upon neurohormonal and mechanical stimulation[40]. Terracio *et al* [38] reported the presence of β1 integrin at Z-discs and at the interface of Z-disc –plasma membrane contact regions, the costameres. From these evidence, it is conceivable that the ECM-costamere-Z-disc axis is important for integrin-mediated signal transduction that governs cellular morphological/growth changes via changing the actin dynamics. For such a function, an actin isoform with 1) distinguishable localization (from the cardiac α−actin isoform) and complex formation with various actin binding proteins, 2) variable polymerization/depolymerization kinetics, and 3) proximity to mechanotransducers such as integrin receptors are necessary. To this end our results suggest that β-actin either alone or in combination with other actin isoform(s) could play a role in the Z-discs and costameres. This notion is further supported by the presence of non-muscle myosin II isoforms in cardiomyocyte Z-discs and intercalated discs [41]. Since NMMIIs are involved in tractional force generation [42], they could be the tangential force generating myosin isoform that works in concert with the β-actin isoform at the costamere-Z-disc axis.

Our data showing an increased contractility upon overexpression of β-actin or decreased contractility in β-actin neutralized with a specific antibody suggests that β-actin filaments promote the contractile force in cardiomyocytes. Based on the localization of β-actin in the present study at the Z-disc-costameric axis and based on the localization of integrins at the costamere, we speculate that the level and polymerization state of β-actin is expected to increase the adhesion force of integrin and ECM. In fact, a recent study using atomic force microscopy has reported that the cardiomyocyte integrin - ECM (fibronectin) adhesion force is determined by cell stiffness as well as the contractile status of the cardiomyocytes [43]. In addition, factors such as increased calcium transients as well as re-expression of other sarcomeric proteins as early compensatory mechanisms to meet the increased work-load and improve cardiomyocyte contractile performance cannot be ruled out. Thus, tension/torsional forces applied onto the integrin-ECM linkage sites during events such as PO, could alter the composition and function of costameric complexes in order to adapt to the changes in contractility and to maintain structural integrity.

To study β-actin's role in cardiomyocyte cytoskeletal organization, we focused on changes in a critical structural element Z-disc, which is known to undergo rearrangement in hypertrophying cardiomyocytes. Our results indeed show that β-actin associated with the Z-discs undergoes dynamic exchange with the cytoplasmic β-actin pool and this rate of exchange is enhanced by hypertrophic agonists. Exchange of proteins from the cytoplasmic pool into the Z bands has been demonstrated using FRAP in skeletal muscle Z-discs [7]. These studies demonstrate that various Z-band proteins such as α-actin, α-actinin, cypher, FATZ, myotilin, and telethonin have different reassembly rates. Myofibrils of skeletal myocytes as proposed by Wang *et al* [7] are maintained by the constant exchange of proteins between these organized structures and the cytoplasmic pool. This exchange allows (i) removal of proteins that are old (for example, the half life of actin is 7.5 days after which the protein molecule has to be substituted with a newly synthesized actin molecule), (ii) insertion of new proteins with altered scaffolding functions for signaling events, (iii) addition of extra molecules into the complex that are required for cell growth, by adding more myofibrils or sarcomeres [44]. Our data is further supported by the finding that endothelin and phenylephrine enhance the dynamics of CapZ, an actin capping protein [45].

A comparative study of actin dynamics using rhodamine labeled actin in actin myofilaments in chick embryonic cardiomyocytes and stress fibers of fibroblasts revealed that the fluorescent recovery in fibroblasts was faster than that in cardiomyocytes [46]. The observed slower dynamics in cardiomyocytes is reflected in the overall structural dynamics of the cell wherein the morphological changes in fibroblasts are dramatic in contrast to a much slower morphological transformation of cardiomyocytes. Further studies with prolonged time points upon hypertrophic stimulation will provide more insight into the process of actin cytoskeletal rearrangement during adult cardiomyocyte growth.

The actin cytoskeleton can be regulated by a range of extracellular stimuli such as growth factors, G-protein coupled receptors, lipids, and integrin-ECM interactions. Of the critical downstream cytoskeleton effectors, many are also involved in hypertrophic responses including the Rho family GTPases, calcium, reactive oxygen species, tyrosine kinases, MAPK, and PKCs. Recently mTOR has also been shown to regulate actin cytoskeleton in other cell types [47] suggesting the mTOR activation observed by us and others in cardiomyocytes during hypertrophy [48] contributes to cytoskeletal rearrangement of adult cardiomyocytes. Since many of these cell signaling pathways converge upon cytoskeletal elements such as β-actin it is conceivable that modulating β-actin dynamics can be a strategy to control tissue remodeling and cardiomyopathy. One example, Rac1, which is involved in mediating cardiomyocyte hypertrophy [49] could affect the dynamics of β-actin isoform at the costameres and Z-discs. Our results show that the expression of dominant negative Rac1 (N17 Rac) in adult cardiomyocytes reduce the ET-stimulated increase in β-actin dynamics. Evidently, Rho family GTPase inhibitors such as statins have been reported to blunt cardiac hypertrophic responses [50] suggesting the suitability of taping β-actin dynamics to modify a tissue phenotype.

In summary, our study suggests that β-actin present in Z-discs and costameres with its polymerization/depolymerization dynamics could govern the tangential force and cytoskeletal recruitment of signaling elements for hypertrophic cellular responses in adult cardiomyocytes.
